# Editorial: Vestibular function and mental health during the lifespan

**DOI:** 10.3389/fneur.2026.1779029

**Published:** 2026-02-04

**Authors:** Kathrine Jáuregui-Renaud

**Affiliations:** Unidad de Investigación Médica en Otoneurología, Instituto Mexicano del Seguro Social, Ciudad de México, Mexico

**Keywords:** anxiety, depression, mental symptoms, tinnitus, vestibular

Neurological disorders can manifest with mental symptoms and decreased performance in certain cognitive domains. These relationships are mediated by widespread sensory networks that are set to provide rapid attention and selection of adaptive actions in response to salient stimuli with emotional influences (1–3). These multisensory connections reflect fundamental survival functions for both internal and external needs, including the vestibular system's influence on preprogrammed sensorimotor patterns of approach or avoidance (4). Emotions and the perception of emotional expressions can prompt adaptive actions; for instance, in mammals, different defense modes map onto manifestations of anxiety, fear, and panic (5). However, in the event of individual traits or the amplification and/or persistence of these responses, mental symptoms can become disruptive for adequate performance of daily life activities.

In this Research Topic, Dunlap et al. described how baseline psychosocial factors reported by adults with dizziness can be related to future measures of activity, participation, and community mobility. The main contributing mental factors in this study were anxiety and depression symptoms, fear avoidance, and catastrophizing beliefs. The authors propose that timely identification of the contributing factors with adequate interventions may improve therapy outcomes. Jáuregui-Renaud et al. showed that spatial anxiety can be related to perceived stress in adults with/without vestibular disease. Their study suggests that spatial anxiety and perspective-taking may contribute to the dizziness-related disability reported by patients with peripheral vestibular disease, while state anxiety and acute stress may affect recovery from peripheral vestibular dysfunction. The authors suggest that the mental symptoms related to acute or persistent disease could contribute to self-imposed limitations on beneficial behaviors for adaptation.

To assess the severity of Postural Perceptual Persistent Dizziness (PPPD), Meletaki et al. evaluated the validity of the French adaptation of the Niigata Questionnaire. PPPD is a functional vestibular condition mainly expressed by subjective manifestations that hinder accurate and early diagnosis. The results showed significant correlations between the questionnaire and the dizziness-related disability with high internal consistency, demonstrating this assessment tool's reliability. The study contributes to our understanding of PPPD symptomatology and assessment.

Zhou et al. compared the clinical characteristics, treatments, and prognoses of middle-aged and elderly patients with Benign Paroxysmal Positional Vertigo (BPPV). BPPV is the most common cause of episodic vertigo in adults, with sudden changes in head position provoking episodes (6). The elderly patients in this study exhibited higher dizziness-related handicap (including in the physical, functional, and emotional domains), decreased body balance, an increased number of falls, and a lower rate of performance of physical therapy maneuvers. However, although physical therapy maneuvers are the most effective treatment for this condition (7), they were rejected by some patients. The authors discussed how fear of falling influences the daily lives of elderly patients and the need to improve counseling to increase acceptance of repositioning maneuvers. Further assessment of specific factors contributing to the disability related to BPPV was conducted by Tang et al. They investigated the independent and interactive effects of insomnia and 25-OH-D levels on the dizziness-related handicap reported by patients with BPPV. Baseline analysis showed that patients with insomnia presented with a less favorable clinical profile, including smoking and alcohol consumption, in addition to high blood pressure and diabetes. After multivariable adjustment, insomnia, lower 25-OH-D levels, and their interaction term were independent predictors of higher dizziness-related disability, with synergy. The authors postulate that dysregulation across the neuroendocrine-immune network may contribute to this link; however, the study's design precludes establishing any causal relationship.

Tinnitus is an unwanted auditory perception in the absence of an apparent acoustic stimulus that can co-occur with vestibular dysfunction. It has been associated with anxiety, depression, and sleep disorders (8). Sometimes, tinnitus can be heard by an examiner (objective tinnitus); however, it is frequently described as a mental symptom when it is heard only by the patient (subjective tinnitus). Zhang, Wang et al. assessed the risk factors associated with tinnitus in individuals with hearing loss, with a special focus on older adults. Their analysis showed that five independent predictors of moderate-to-severe tinnitus among patients with hearing impairment were older age, more severe hearing loss, decreased sleep quality, anxiety symptoms, and blood hypertension. The authors propose screening for sleep quality and anxiety in the assessment of patients with hearing loss. In a second study, Zhang, Ran et al. developed and validated a clinical prediction model for moderate-to-severe tinnitus in patients with hearing loss. The analysis showed that hearing loss severity, age, and sleep quality were the most influential predictors. Additionally, the analysis corroborated that tinnitus severity was related to depression and anxiety. Their results suggest that integrating auditory and psychological factors could improve tinnitus risk identification in patients with hearing impairment.

**Figure 1:**
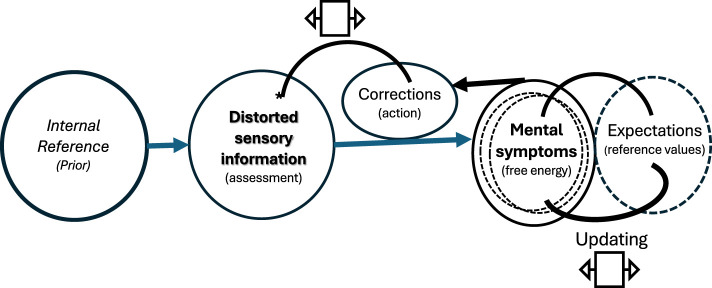
Simplified representation of the mental consequences of sensory dysfunction, according to the predictive coding and action inference theory (10). Dysfunctional sensory inputs may evoke inadequate corrections and erroneous assessment of their consequences, increasing the variation of the discrepancy between the predicted and the observed information, and hindering the updating of the expectations.

Vestibular information intermingles with other sensory inputs in a variety of brain processes that drive action control, including mental displays. In the context of predictive coding and active inference theory (9, 10), the results of the studies included in this Research Topic support the idea that sensory misinformation may disrupt both the physiological processes supported by the sensory input and the feedback required to mitigate the effects of dysfunction through suitable countermeasures. Unless the sensory function is restored or substituted, the imprecise sensory inputs may increase the variability of the discrepancy between predictions and observations (free-energy variation), including those of the predicted effects of any countermeasure. This undesirable outcome could be amplified by individual traits and inadequate corrections, while hindering adaptation through updating expectations based on experience (Figure 1). From this perspective, when the potential for sensory restoration/substitution decreases, rehabilitation strategies could promote sensory reweighting for integration, while individual counseling could facilitate personalized interventions to mitigate the consequences of mental symptoms generated by sensory dysfunction.

Further studies are required to assess the underlying mechanisms of the mental consequences of sensory dysfunction in humans and to understand the mental effects of both sensory stimulation and sensory disorders, particularly those related to vestibular function in health and disease.
